# Implementation of Spirometry Telemonitoring Programme in Lung Transplant Recipients: A Retrospective, Controlled Analysis of Clinical Outcomes

**DOI:** 10.3390/life16081224

**Published:** 2026-07-24

**Authors:** Mikołaj Basza, Wojciech Bojanowicz, Fryderyk Zawadzki, Dagmara Galle, Mateusz Soliński, Weronika Kowalczyk, Łukasz Kołtowski, Marek Ochman

**Affiliations:** 1 Department of Cardiac, Vascular and Endovascular Surgery and Transplantology, Medical University of Silesia in Katowice, 41-800 Zabrze, Poland; mikolaj.basza@wum.edu.pl (M.B.); wojtekbojanowicz@gmail.com (W.B.); d201247@sum.edu.pl (D.G.); 2 Department of Pulmonology and Internal Medicine, W E. J. Zeyland Wielkopolska Center of Pulmonology and Thoracic Surgery, 60-569 Poznan, Poland; 3 School of Biomedical Engineering & Imaging Sciences, Faculty of Life Sciences & Medicine, King’s College London, Strand, London WC2R 2LS, UK; mat.solinski@gmail.com; 4 Engineering Department, Faculty of Natural, Mathematical & Engineering Sciences, King’s College London, Strand, London WC2R 2LS, UK; 5 1st Department of Cardiology, Medical University of Warsaw, 02-091 Warsaw, Poland; werakowalczyk@wp.pl (W.K.); lukasz.koltowski@wum.edu.pl (Ł.K.); 6 Department of Thoracic Surgery, Poznan University of Medical Sciences, 60-569 Poznan, Poland; mochman@wcpit.org

**Keywords:** lung transplantation, spirometry, telemedicine

## Abstract

**Background:** Lung transplantation (LTx) is the final therapeutic option for patients with end-stage irreversible respiratory failure. Chronic lung allograft dysfunction (CLAD) remains a major determinant of long-term outcomes, but early detection of functional decline may enable timely intervention and partial reversibility. Therefore, systematic monitoring of graft function is essential, and telemedicine-based spirometry may facilitate early identification of clinically relevant decreases in lung function. **Objective:** The aim of our study was to evaluate the feasibility of spirometry telemonitoring (TM) and its association with healthcare utilisation in lung transplant patients. **Methods:** This retrospective study compared lung transplant recipients: the first group underwent spirometry TM (*n* = 21), the second group was monitored with standard home spirometry (HS) (*n* = 23), and the control group underwent routine follow-ups only at the transplant centre (*n* = 32). **Results:** In the TM spirometry group, mean adherence was 62.7%, with 4957 examinations performed over a mean monitoring period of 252 days; 59.7% of FEV1 and 58.9% of FVC measurements were technically acceptable. Compared with routine care (RC), TM spirometry was associated with a shorter mean duration of both combined planned and urgent admissions (5.8 vs. 10.0 days, *p* < 0.001) and urgent admissions analysed separately (14.7 vs. 26.2 days, *p* < 0.001). **Conclusions:** TM spirometry was feasible and associated with lower healthcare utilisation in lung transplant recipients, supporting its potential value for post-transplant surveillance. Larger prospective randomised studies are needed to confirm these preliminary findings.

## 1. Introduction

Lung transplantation (LTx) is the definitive treatment for selected patients with end-stage irreversible respiratory failure. Although the number of procedures continues to increase worldwide, with approximately 4500 lung transplants performed annually, LTx remains resource-intensive and costly [1,2]. Candidates include patients with advanced chronic obstructive pulmonary disease, interstitial lung disease, cystic fibrosis, and idiopathic pulmonary arterial hypertension, in whom transplantation is often the only remaining therapeutic option [3,4,5,6,7,8,9]. Given the scarcity of donor lungs, substantial effort is devoted to candidate selection and organ allocation to ensure the greatest possible individual and population-level benefit [4]. 

However, maintaining this benefit after transplantation is equally important and depends on rigorous post-transplant surveillance, as lung transplantation remains associated with a substantial risk of complications. Post-operative issues include infections related to intensive immunosuppression, acute and chronic graft rejection, airway remodelling, neoplastic changes, and increased risk of gastroesophageal reflux [3]. One of the most critical long-term complications is chronic lung allograft dysfunction (CLAD), which affects more than half of recipients within five years [10]. CLAD is the leading cause of graft failure and long-term mortality after LTx. It is characterised by a persistent decline in forced expiratory volume in 1 s (FEV1), detected through spirometry. CLAD manifests in various phenotypes, obstructive, restrictive, or mixed, and is multifactorial in origin. Chronic rejection is a major determinant of long-term outcomes after lung transplantation, occurring in up to 70% of patients within 10 years and sometimes as early as three months post-surgery [11]. It most commonly manifests as bronchiolitis obliterans syndrome (BOS), a progressive obstructive condition defined by irreversible FEV1 decline due to bronchiolar fibrosis and obliteration, ultimately leading to respiratory failure [12,13].

Early detection of lung function decline is therefore central to post-transplant surveillance. Spirometry, particularly FEV1, remains the main tool for detecting BOS and monitoring graft function [14,15,16,17,18,19,20,21,22,23,24]. Home monitoring (HM) can identify deterioration before symptoms develop, but adherence is a major limitation. Poor health status, low motivation, time constraints, and limited internet access contribute to reduced compliance [25,26,27]. Adherence appears highest during the first post-transplant year, in older recipients, and in patients with BOS, whereas younger patients, particularly those with cystic fibrosis, are less consistent in performing HM [15].

Real-time clinician access to home measurements may improve adherence, and targeted education can further support appropriate spirometry use [25,26]. A ≥10% decline in FEV1 compared with baseline should prompt diagnostic work-up and clinical evaluation according to ISHLT guidance [10]. However, standard HM depends on patient competence, manual recording, and patient-initiated contact, while test quality and patient-reported data may vary. Early detection may have clinical and economic relevance, as early-stage CLAD management is less costly than treatment of advanced disease [17]. These limitations have led to growing interest in digital and computer-based monitoring systems that enable real-time analysis of lung function and patient-reported data [21,23]. Such tools may support more efficient surveillance, particularly as transplant volumes increase.

Recent ISHLT definitions introduced acute lung allograft dysfunction (ALAD), defined as a ≥10% decline in FEV1 from the maximum value within the preceding 180 days [28]. ALAD captures acute and potentially reversible graft dysfunction, including infection, acute rejection, and antibody-mediated rejection [29,30]. Because persistent or progressive ALAD is associated with substantially increased graft-loss risk, frequent spirometric monitoring is clinically relevant for early detection and timely intervention [28].

In this context, HM and telemonitoring spirometry (TM) represent distinct surveillance strategies. Standard HM relies on patient-recorded measurements and patient-initiated contact, whereas spirometry TM enables automatic data transmission, clinician access to longitudinal trends, and potentially earlier response to relevant lung function decline. However, real-world comparative data on spirometry TM versus standard HM and routine centre-based follow-up remain limited. We therefore hypothesised that spirometry TM would be feasible and associated with lower healthcare utilisation. This study evaluated the feasibility of a structured spirometry TM programme and compared selected healthcare utilisation outcomes among lung transplant recipients managed with TM, standard HM, or routine care.

## 2. Materials and Methods

### 2.1. Study Design

This was an investigator-initiated, observational, retrospective, non-interventional, non-randomised study. The study conforms to the ethical guidelines of the 1975 Declaration of Helsinki and was acknowledged by the Institutional Board Review Committee on Human Research of the Medical University of Silesia in Katowice (registration number: BNW/NWN/0052/KB/47/24). The patients who underwent LTx in the Silesian Centre for Heart Diseases (Zabrze, Poland) between December 2021 and July 2022 were equipped with the portable spirometer (AioCare, HealthUp, Warsaw, Poland). They were trained by an experienced operator on discharge from the hospital to perform spirometry examinations at home, in line with American Thoracic Society/European Respiratory Society (ATS/ERS) guidelines [25]. They were asked to perform daily spirometry tests and report respiratory symptoms using the mobile app (AioCare Patient, HealthUp, Warsaw, Poland). The observation period for the spirometry TM group started at discharge after lung transplantation and continued for the available follow-up period. All examinations were transmitted in real time to the web platform (HealthUp, Warsaw, Poland) and reviewed by the attending physician. Clinically relevant declines in spirometry parameters or reported symptoms could trigger patient contact, teleconsultation, or urgent hospital assessment. During planned admissions, TM data were also used to assess longitudinal trends in lung function. The number of examinations, technical acceptability, adherence to the protocol, and spirometry results were evaluated. Additionally, the number and length of emergency and elective admissions were compared between the TM group and the comparison groups.

### 2.2. Equipment and Usage

Spirometry tests were conducted using the portable spirometer (AioCare^®^, HealthUp, Poland). This device is classified as a class IIa hospital-grade equipment. It pairs with a patient’s smartphone (iOS/Android) through Bluetooth^®^ and is managed by the AioCare Patient mobile app. Patients were required to register an account and enter their biometric information (age, birth sex, ethnicity, weight, and height) and fundamental medical information. The app instantly assessed the technical quality of spirometry tests according to the ATS/ERS 2019 Standards [31]. All collected data were automatically uploaded to a secure health cloud platform (AioCare Doctor), enabling doctors to assess the examinations and trends in real time.

### 2.3. Comparison Groups

The spirometry TM group (TM group) included patients who underwent lung transplantation between December 2021 and July 2022 and were enrolled during the implementation of the TM programme. Results from the TM group were retrospectively compared with two groups of lung transplant recipients managed at the same centre between 2017 and 2022: a standard HM group (HS group) and a routine care (RC) group. Group allocation was non-randomised and reflected real-world monitoring strategies used in routine post-transplant care across different time periods. Patients in the HS group were equipped with a portable spirometer (MIR, Italy) at discharge, educated on HM, instructed to keep a daily spirometry diary, and advised to contact the attending physician if spirometry parameters decreased significantly. Patients in the RC group did not use digital monitoring or structured HM. All groups remained under routine transplant-centre care according to ISHLT recommendations [4,10,11].

### 2.4. Analysis

Demographic and spirometry data are presented as means with 95% confidence intervals or ranges, as appropriate. Continuous variables were compared using ANOVA or the Kruskal–Wallis test, depending on distributional assumptions. Normality was assessed separately within each group using the Kolmogorov–Smirnov test, and ANOVA was applied only when all groups showed a normal distribution. Categorical variables were analysed using the chi-square test, with Yates’ continuity correction applied when expected cell counts were <5. Statistical significance was set at *p* < 0.05. In the TM group, adherence, technical acceptability of spirometry examinations, defined as grade A or B according to ATS/ERS 2019 criteria, and mean percentage-predicted spirometry values (%predFEV1, %predFVC, %predFEV1/FVC) were presented monthly. Temporal trends were assessed using fitted trend lines and Pearson correlation coefficients.

Hospital admissions were retrospectively identified from electronic health records, classified as planned or urgent, and measured from admission to discharge. Urgent admissions were defined as unscheduled hospitalisations due to clinical deterioration, respiratory symptoms, abnormal spirometry, or other acute concerns. The number of admissions, including all, planned, and urgent admissions, and their mean and maximum duration were compared between groups (with the RC group used as the reference category), using linear mixed models adjusted for age, sex, and monitoring duration (in years), with random intercepts for the year of transplantation. Model diagnostics included visual inspection of residual-versus-fitted plots and Q–Q plots of residuals to assess the influence of individual observations (the results of the diagnostics were shown in the Supplementary Materials). Analyses were performed in MATLAB 2022b. No formal a priori sample size calculation was performed because this was a retrospective analysis of all available patients; therefore, analyses should be considered exploratory.

## 3. Results

Twenty-one patients after lung transplantation (nine females and 12 males) were recruited to the study, with a mean age of 53.0 (95%CI: 47.3–58.6) years and a mean body mass index (BMI) of 21.4 (95%CI: 19.5–23.2) kg/m^2^. The summary of the demographic data is shown in Table 1.

The average time of monitoring of each patient was 252.2 (min: 53, max: 445) days with a mean adherence level (following the rule: 1 spirometry per day) of 62.7% (min: 15.1%, max: 97.4%). The mean number of spirometry examinations was 225 (min: 9, max: 806) per patient; among these, the mean percentages of measuring acceptable FEV1 and forced vital capacity (FVC) were 59.7% (95%CI: 44.7–74.6%) and 58.9% (44.2–73.6%), respectively. The total number of examinations performed by all patients was 4957 (15,875 single manoeuvres). The summary of the performance parameters for each participant is presented in Table S1.

**Figure 1:**
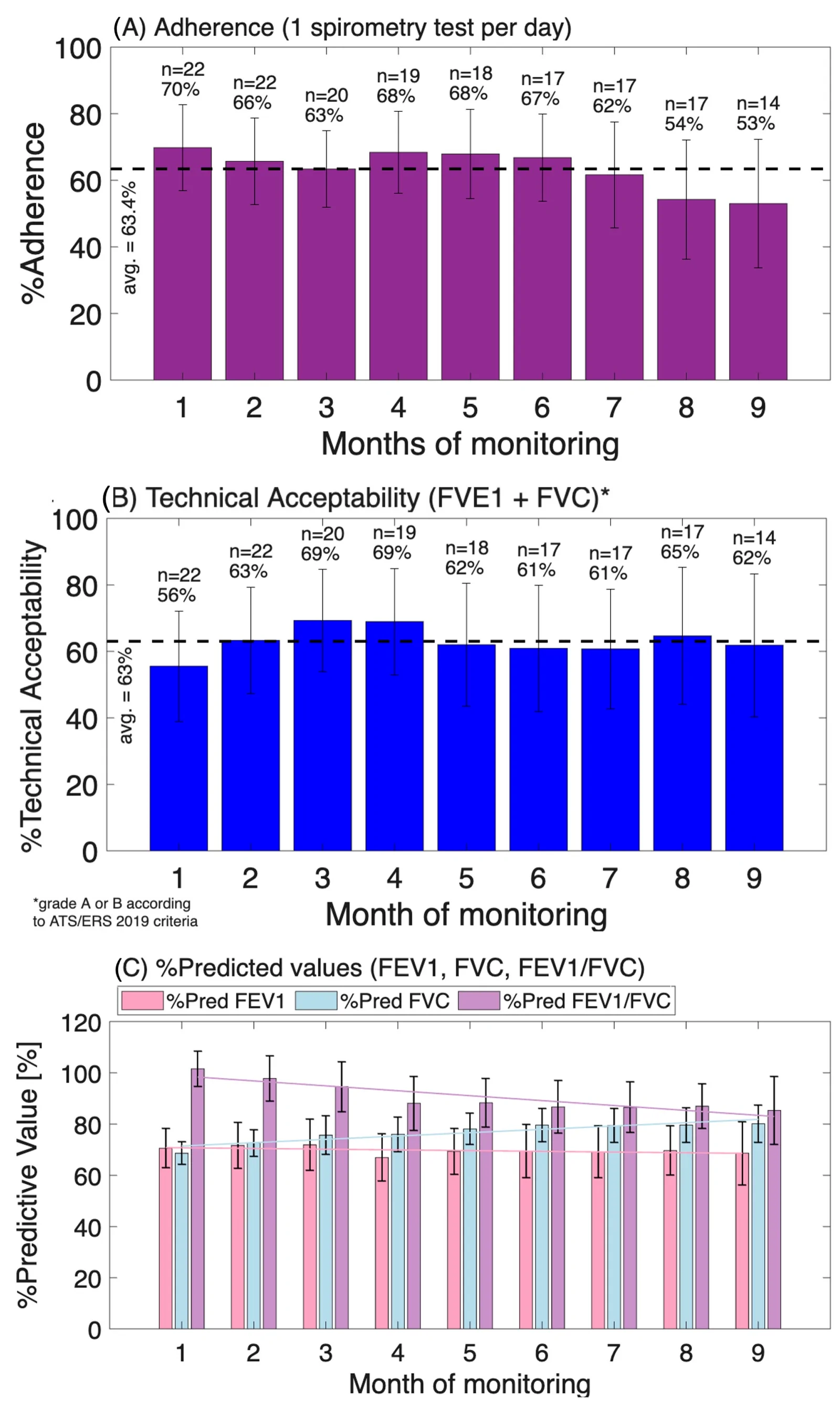
Monthly adherence, acceptable spirometry examinations, and %Predicted FEV1, FVC, and FEV1/FVC during months 1–9 of monitoring, with percentages and participant numbers shown above the bars.

The percentage adherence, technical acceptability level, and spirometry results within each month of monitoring are presented in Figure 1.

**Figure 2:**
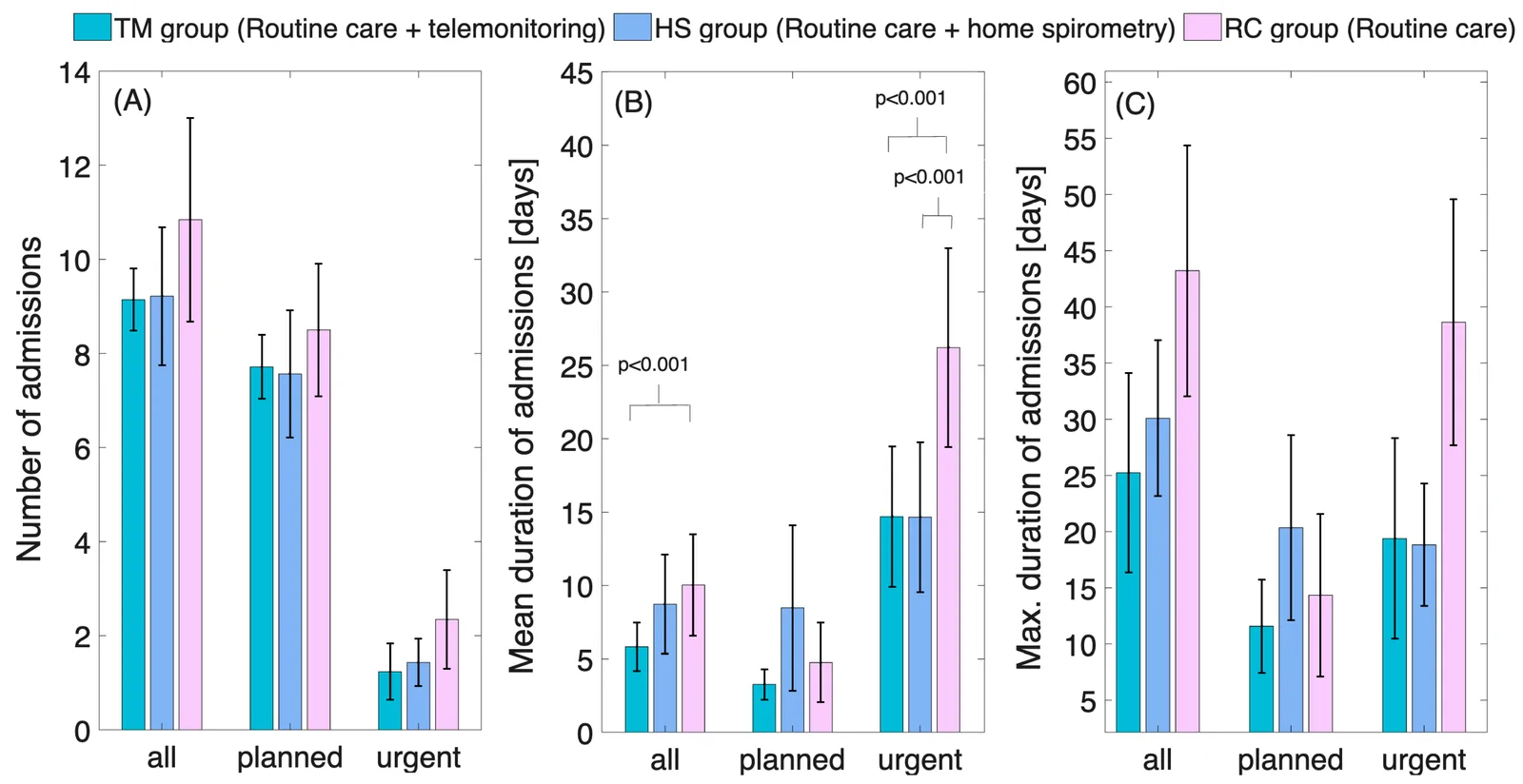
Comparison results of (**A**) the number of admissions, (**B**) the mean duration of admissions, and (**C**) the maximum duration of admissions between three groups: routine care + telemonitoring, routine care + home monitoring, and routine care. The comparisons were calculated for all, only routine, and only emergency admissions.

We observed relatively stable adherence during the first 6 months (63–70%; Figure 1A). Thereafter, adherence declined over the following 3 months, falling below the overall average (53–62%). The proportion of technically acceptable spirometry examinations was lower during the first month of monitoring (56%) and increased to 69% in months 3 and 4. From month 5 onward, examination technical acceptability remained close to the overall average, ranging from 61% to 65% (overall mean 63%; Figure 1B). Figure 1C presents the mean %Predicted values of FEV1, FVC, and FEV1/FVC for each monitoring month. A significant increase in %Predicted FVC and a significant decrease in %Predicted FEV1/FVC were observed over time, with Pearson correlation coefficients of r = 0.94 (*p* < 0.001) and r = −0.90 (*p* < 0.001), respectively. A decrease in %Predicted FEV1 was also observed, although it did not reach statistical significance (r = −0.50, *p* = 0.17). The comparison of admission frequency, mean admission duration, and maximum admission duration between study groups is presented in Figure 2.

Mixed-effects models demonstrated that the mean duration of all admissions was significantly longer in the RC group (10.0 (6.6–13.5) days) than in the TM group (5.8 (4.2–7.5) days; β = −7.1 (−10.8–−3.3), *p* < 0.001). Similarly, the mean duration of the urgent admissions was significantly longer in the RC group (26.2 (19.4–33.0) days) than in TM (14.7 (9.6–19.8) days, β = −15.1 (−22.9–−7.3), *p* < 0.001) and HS (14.7 (9.9–19.5) days, β = −14.2 (−21.7–−6.7), *p* < 0.001); these differences remained significant after considering the Bonferroni correction for nine comparisons: three outcome parameters across three visit categories (adjusted significance threshold: *p* < 0.0056). Effect sizes for all models are presented in Supplementary Tables S2–S10. Within the TM group, adherence was not significantly correlated with the number of urgent admissions (r = 0.230), mean urgent admission duration (r = −0.049), or maximum urgent admission duration (r = 0.274).

## 4. Discussion

We evaluated the technical and healthcare utilisation outcomes of spirometry TM in lung transplant recipients at the Silesian Centre for Heart Diseases in Zabrze and assessed its feasibility for routine post-transplant follow-up. Several home-monitoring strategies for early BOS detection after lung transplantation have been reported, differing in workflow, reporting structures, and the degree of patient–physician interaction [14,15,16,17,18,19,20,21,22,23,24]. Despite these difficulties, maintaining long-term patient adherence remains one of the principal challenges for the successful implementation of spirometry TM programmes.

Reported adherence rates vary considerably across studies. The study by Fadaizadeh et al. examined lung transplant patient adherence to HM during the first six months after hospital discharge. They observed a high 80% adherence rate in one patient, while another showed a still-respectable 61% adherence despite some punctuality issues, likely due to internet connectivity problems [24]. Morlion et al. reported adherence rates of 55% for twice-daily measurements and 84% for daily measurements [22], whereas Finkelstein et al. observed 82% adherence for weekly spirometry submissions [21]. In a larger cohort of 287 lung transplant recipients followed for a mean of 54 ± 45 months after transplantation, Kugler et al. found that 43% of patients did not adhere to the prescribed home-monitoring protocol [15]. In our study, adherence averaged 63.4% over a nine-month period, placing it within the range reported in previous studies. We also observed a gradual decline in adherence over time.

This adherence pattern is consistent with the findings of Liu et al., who demonstrated that lung transplant recipients more than one year post-transplant had substantially lower odds of engaging with an mHealth spirometry programme (OR 0.16, 95% CI 0.07–0.35), while non-English-speaking patients also showed significantly lower participation rates (OR 0.22, 95% CI 0.07–0.67) [32]. Qualitative interviews in that study identified several barriers to sustained engagement, including technical difficulties, communication challenges between patients and healthcare providers, a desire for more personalised feedback, and limited understanding of how spirometric data are used in clinical decision-making [32]. Additionally, our study noted a high level of technical accuracy in spirometry tests conducted at home by patients (average 63%), which remained consistent throughout the nine-month tracking period.

Only a few studies, similarly to our approach, examined the clinical implications of spirometry TM combining telemedical and digital tools in this group of patients [19,23]. Odisho [23] showed that using the proposed chat-based spirometry alerting system, they detected 15 cases of CLAD and seven cases of CLAD progression through HM alerting. Patient cost-saving analysis was measured as the number of admissions reduced during the 2-year follow-up and transportation costs for the 155 patients transplanted since the beginning of the program; this amounts to a total of $179,899.20. The authors did not present a detailed analysis of the reduced number of admissions or a cost-effectiveness analysis for healthcare providers. Therefore, direct comparison was impossible. In this retrospective cohort, spirometry TM was associated with a shorter mean duration of all admissions and urgent admissions analysed separately compared with RC. However, because of the retrospective, non-randomised design, limited sample size, and potential residual confounding, these findings should be interpreted as exploratory and hypothesis-generating rather than evidence that TM caused shorter hospital stays. Moreover, hospital admission frequency and duration are indirect healthcare utilisation measures rather than transplant-specific clinical endpoints and may be influenced by centre-level practices, clinical workflows, admission thresholds, bed availability, pandemic-related organisational changes, temporal changes in post-transplant care, or unmeasured baseline characteristics. Therefore, the observed differences may reflect the telemonitoring strategy, but they should not be interpreted as evidence of improved graft-related clinical outcomes or attributed to the TM intervention alone. It is worth noting that in the Odisho study, the inclusion criteria differed, and the median time since transplant was 5.3 and 1.9 years, respectively, for the engaged (>=1 submitted FEV1 via chat) and non-engaged groups. Patients in our study were recruited at hospital discharge and monitored for the first 9 months after the transplant, when complication risk is the highest. Our findings align with Odisho that the engagement of patients, and therefore adherence and technical acceptability, may be higher in the first years after the lung transplant. Similar to our findings, Sengpiel observed no significant differences in the numbers of emergency and routine admissions. Sengpiel compared home monitoring to spirometry TM in a Randomised Controlled Trial. Nevertheless, a trend toward shorter intervals between symptom onset and time to consultation was present. Although our study showed a significant difference only in the mean duration of all admissions between the two approaches, further randomised controlled trials should carefully investigate the impact of telehealth interventions in this patient population, with a focus on standardising TM and the digital intervention protocol. Importantly, early post-transplant spirometry may reflect not only graft function but also recovery of respiratory muscle strength—diaphragmatic dysfunction persists up to 6 months after bilateral lung transplantation, and FEV1 correlates closely with diaphragm thickening ratio (r = 0.81) [33,34]—as well as general deconditioning. Additionally, daily spirometry may reinforce self-management engagement; Rosenberger et al. showed that self-monitoring during the first post-transplant year was associated with reduced mortality (HR 0.45), while reporting abnormal findings to clinicians was associated with lower BOS risk (HR 0.27) [35].

### Limitations

This study has several limitations. First, it was a retrospective, non-randomised analysis of a spirometry TM programme implemented during the COVID-19 pandemic; therefore, causal inference cannot be made, and the findings should be considered exploratory. Second, the sample size was limited, particularly in the TM group (*n* = 21), and no formal a priori sample size calculation was performed, limiting statistical power for marginal comparisons, subgroup analyses, and clinical outcomes. Third, although baseline characteristics were comparable, the groups were not randomised and reflected real-world monitoring strategies used across different time periods; the RC group included a numerically higher proportion of patients transplanted for pulmonary hypertension, which may have influenced recovery trajectory, spirometry performance, and healthcare utilisation. Moreover, residual confounding and temporal bias cannot be excluded. Although multivariable adjustment was applied, the absence of more advanced balancing methods, such as propensity score matching or weighting, should be considered when interpreting the comparative analyses. In addition, adherence and technical acceptability were available only for the TM group, and standard HM was not systematically validated against in-hospital spirometry. Finally, CLAD progression, graft function, survival, rehabilitation intensity, muscle strength, treatment adherence and cost-effectiveness were not systematically assessed.

## 5. Conclusions

In this retrospective controlled analysis, spirometry TM was feasible in lung transplant recipients, with good adherence and a high proportion of technically acceptable examinations. It was associated with shorter hospital admissions compared with routine follow-up. These preliminary findings should be confirmed in larger prospective randomised studies.

## Figures and Tables

**Table 1:** Group characteristics.

Parameter	TM: Routine Care + Telemonitoring	HS: Routine Care + Home Spirometry	RC: Routine Care	*p*-Value
N	21	23	32	-
Age [y]	53.0 (95%CI: 47.3–58.6)	54.2 (95%CI: 49.0–59.3)	53.7 (95%CI: 49.3–58.1)	0.96
Sex	F: 9, M: 12	F: 9, M: 14	F: 14, M: 18	0.94
Time of monitoring (per person) [y]	1.02 (95%CI: 0.94–1.11)	1.15 (95%CI: 0.84–1.47)	1.46 (95%CI: 1.17–1.74)	0.09
COPD	10 (47.6%)	11 (47.8%)	13 (40.6%)	0.82
Pulmonary fibrosis	7 (33.3%)	9 (39.1%)	9 (28.1%)	0.68
Pulmonary hypertension	1 (4.8%)	2 (8.7%)	6 (18.7%)	0.26
Others	3 (14.3)	1 (4.4%)	4 (12.5%)	0.51

## Data Availability

The data presented in this study are available on request from the corresponding author due to privacy restrictions.

## Supplementary Materials

The following supporting information can be downloaded at: https://www.mdpi.com/article/10.3390/life16081224/s1, Figure S1: Spirometry measurements results (FEV1, FVC, FEV1/FVC) from each patient; Table S1: Summary of the performance of each patient for the telespirometry monitoring group; Table S2: Number of visits–All visits; Table S3: Number of visits–Planned visit; Table S4: Number of visits–Urgent visit; Table S5: Mean duration of visits–All visits; Table S6: Mean duration of visits–Planned visit; Table S7: Mean duration of visits–Urgent visit; Table S8: Max duration of visits–All visits; Table S9: Mean duration of visits–Planned visit; Table S10: Mean duration of visits–Urgent visit.

## Abbreviations

ATS	American Thoracic Society
ALAD	Acute Lung Allograft Dysfunction
BMI	Body Mass Index
BOS	Bronchiolitis Obliterans Syndrome
CLAD	Chronic Lung Allograft Dysfunction
COPD	Chronic Obstructive Pulmonary Disease
ERS	European Respiratory Society
FEV1	Forced Expiratory Volume in 1 s
FVC	Forced Vital Capacity
HM	Home Spirometry
ISHLT	International Society for Heart and Lung Transplantation
LTx	Lung Transplantation
RC	Routine Care
TM	Telemonitoring
