# Supplementary material for: Implementation of Spirometry Telemonitoring Programme in Lung Transplant Recipients: A Retrospective, Controlled Analysis of Clinical Outcomes
Source: Life (Basel). 2026 Jul 24;16(8):1224. doi: 10.3390/life16081224 (PMC13514497; doi:10.3390/life16081224)
Supplement: Supplementary file 1 [file life-16-01224-s001.zip › life-4254944-supplementary.pdf]

Table S1: Summary of the performance of each patient for the telespirometry monitoring group.

ID	Age [y]	Sex [F - female, M - male]	BMI [kg/m^2]	Days of monitoring [days]	Number of examinations	Adherence [%]	Correctness level [%]	Percent of obstruction [%]
1	56	M	27.0	445	322	63.1	13.4	53.5
2	49	F	16.8	409	686	83.1	57.9	26.7
3	58	F	19.8	156	48	29.5	4.2	0.0
4	20	F	16.2	216	174	69.0	83.3	41.4
5	44	F	17.9	371	359	72.5	68.5	51.2
6	40	M	22.7	287	147	51.2	95.9	1.4
7	32	M	17.9	343	806	97.4	27.0	62.8
8	39	M	25.7	244	140	48.0	49.3	24.6
9	54	M	22.0	305	179	56.4	93.9	41.1
10	60	F	26.0	318	315	91.2	69.8	0.0
11	61	M	24.4	98	47	38.8	14.9	28.6
12	64	M	20.1	298	252	72.5	74.2	10.7
13	65	M	27.0	270	187	59.6	37.4	0.0
14	59	F	30.1	291	266	78.0	79.3	49.8
15	68	M	23.9	288	126	40.3	60.3	0.0
16	44	M	15.4	53	9	15.1	11.1	0.0
17	65	M	19.8	56	35	55.4	2.9	0.0
18	66	F	20.3	78	68	78.2	73.5	0.0
19	53	F	14.0	260	166	59.2	94.6	1.3
20	66	M	23.7	263	251	93.9	94.8	5.0
21	53	F	17.2	214	128	54.7	93.8	35.0

Figure S1: Spirometry measurements results (FEV1, FVC, FEV1/FVC) from each patient.

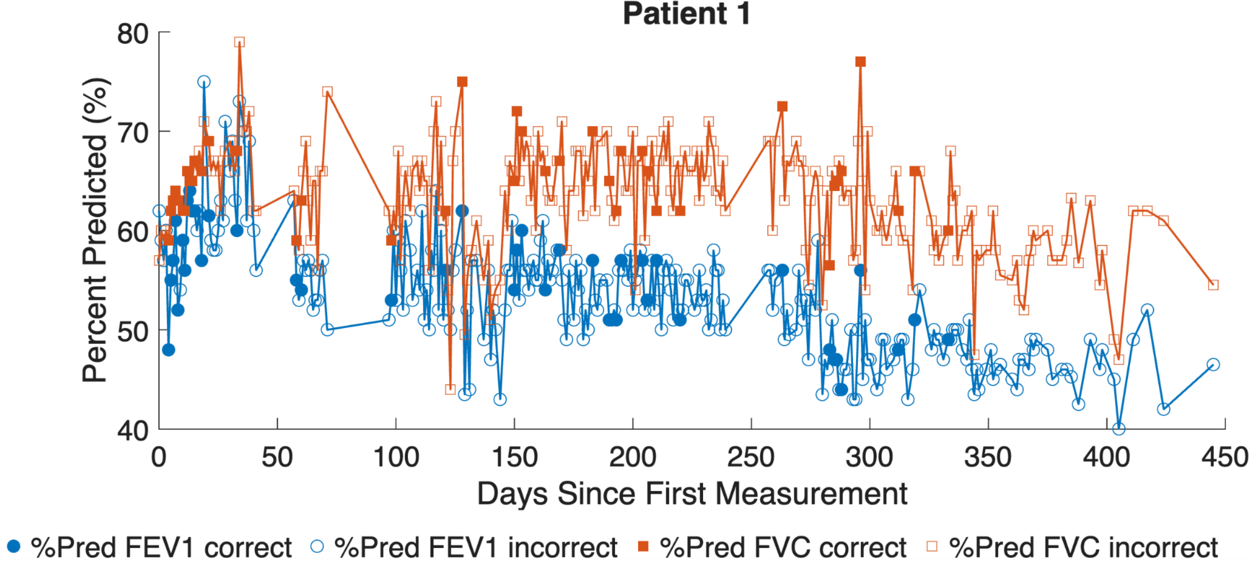
Patient 1
Percent Predicted (%)
Days Since First Measurement
%Pred FEV1 correct
%Pred FEV1 incorrect
%Pred FVC correct
%Pred FVC incorrect

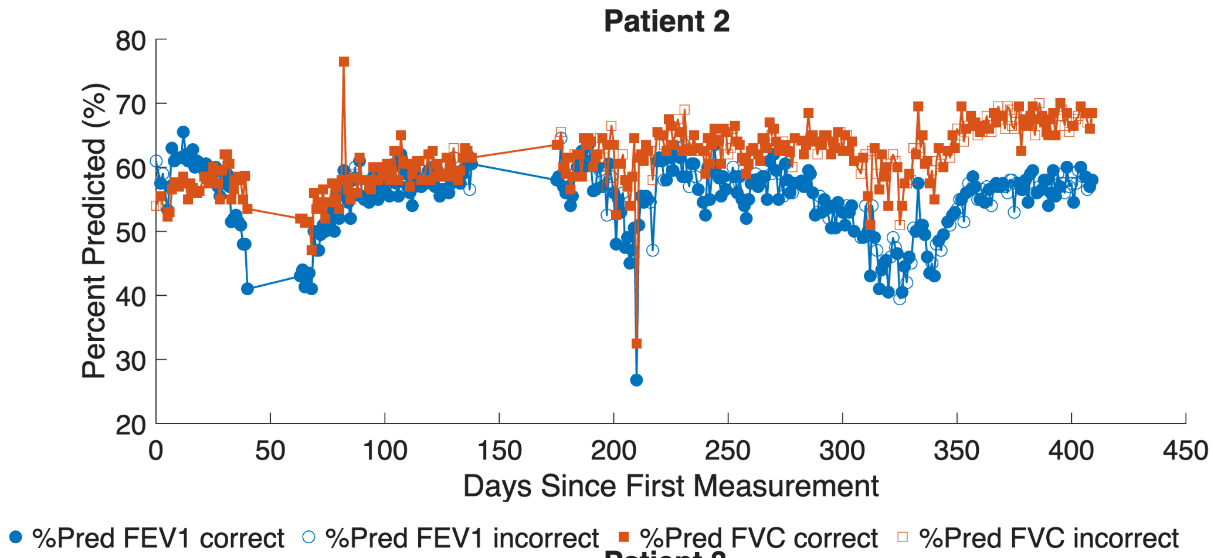
Patient 2
Percent Predicted (%)
Days Since First Measurement
%Pred FEV1 correct
%Pred FEV1 incorrect
%Pred FVC correct
%Pred FVC incorrect

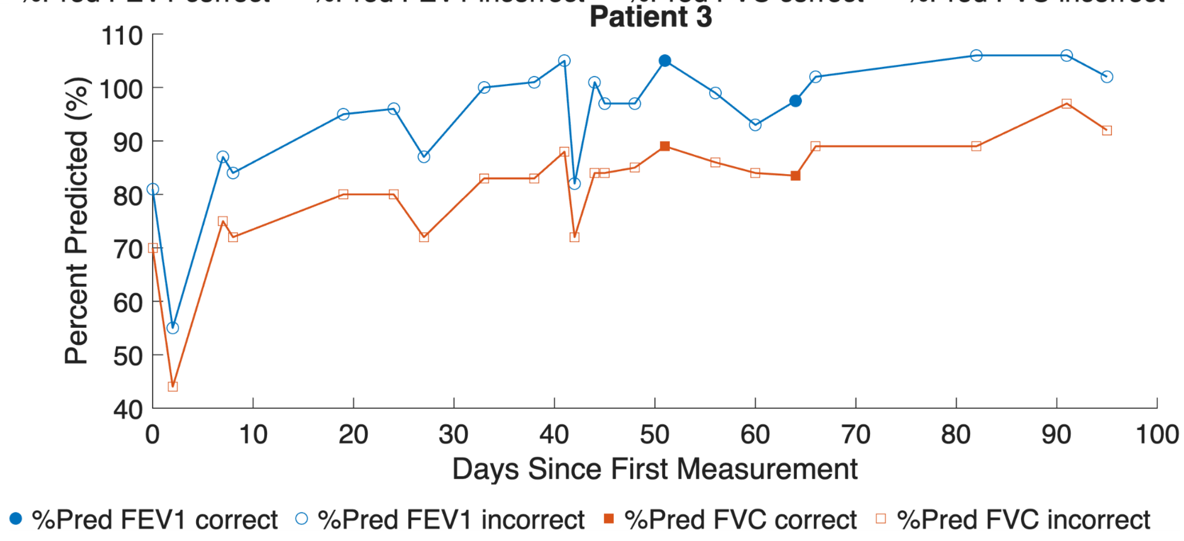
Patient 3
Percent Predicted (%)
Days Since First Measurement
%Pred FEV1 correct
%Pred FEV1 incorrect
%Pred FVC correct
%Pred FVC incorrect

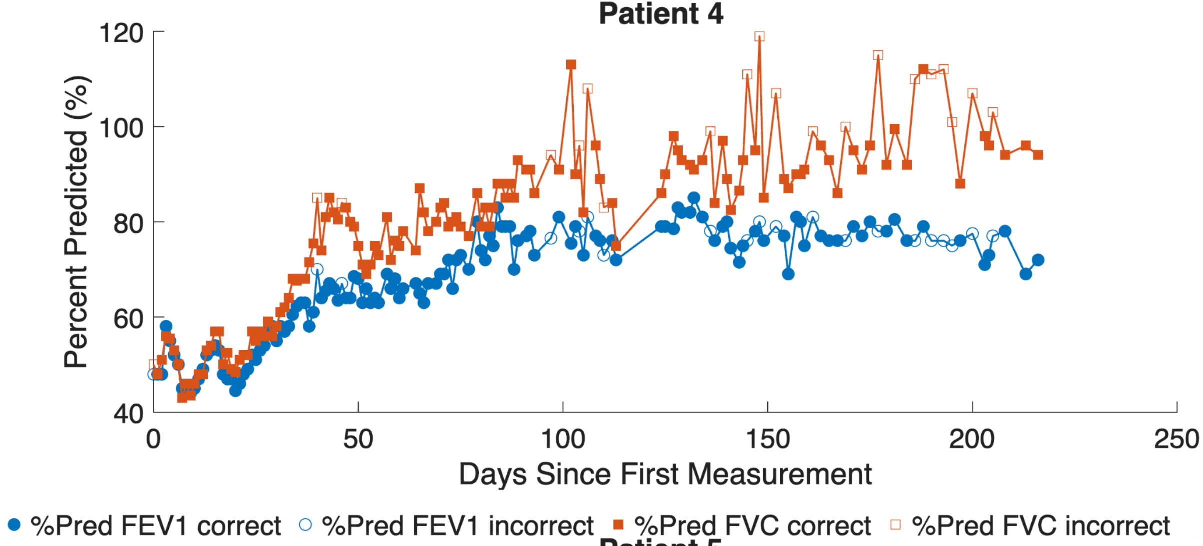
Patient 4
Percent Predicted (%)
120
100
80
60
40
0
50
100
150
200
250
Days Since First Measurement
%Pred FEV1 correct
%Pred FEV1 incorrect
%Pred FVC correct
%Pred FVC incorrect

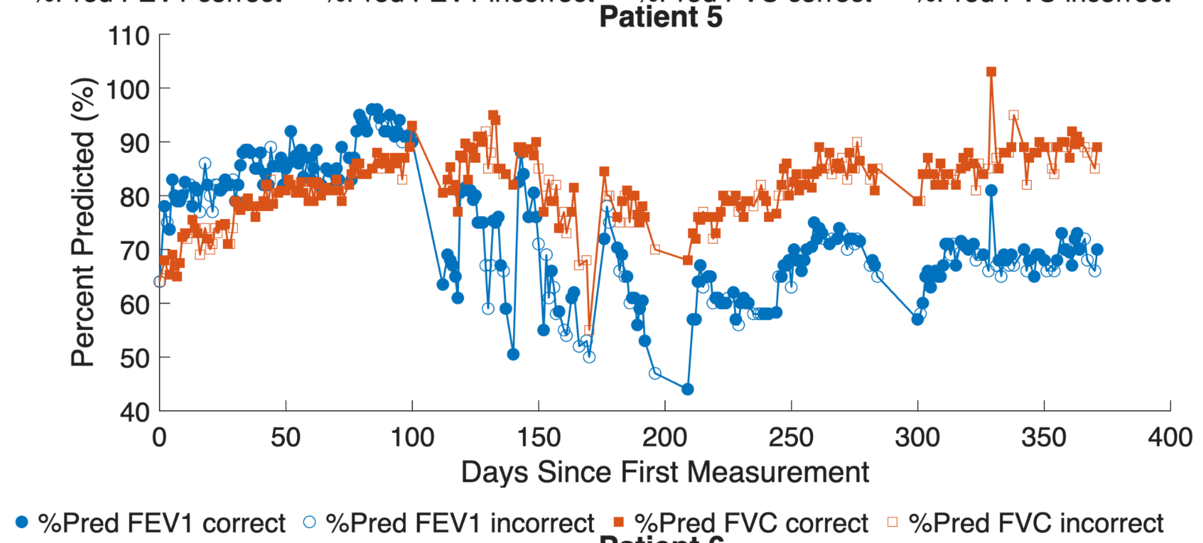
Patient 5
Percent Predicted (%)
110
100
90
80
70
60
50
40
0
50
100
150
200
250
300
350
400
Days Since First Measurement
%Pred FEV1 correct
%Pred FEV1 incorrect
%Pred FVC correct
%Pred FVC incorrect

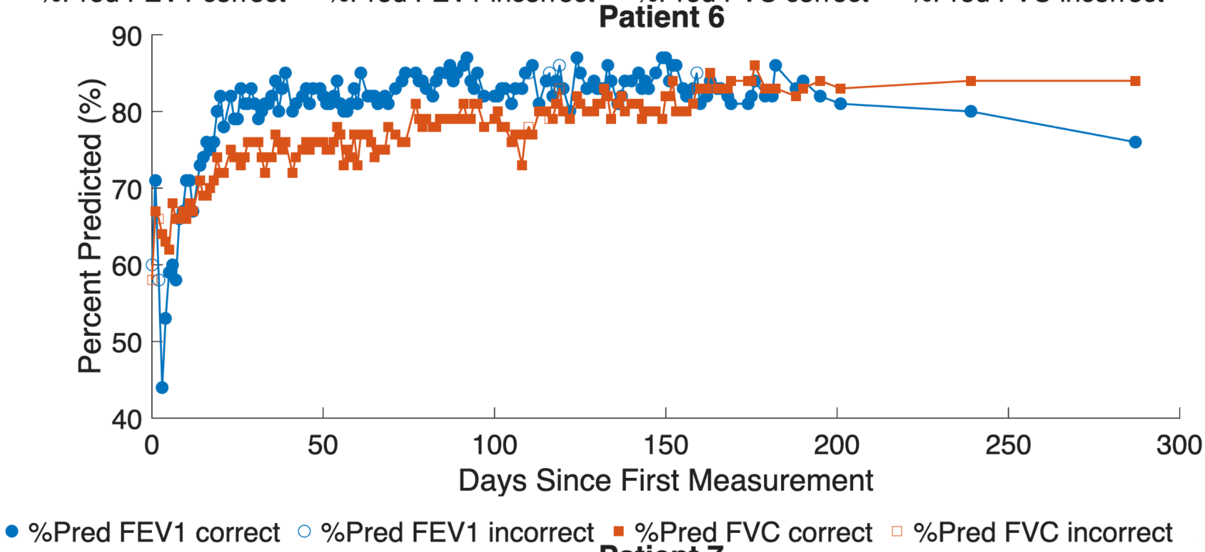
Patient 6
Percent Predicted (%)
90
80
70
60
50
40
0
50
100
150
200
250
300
Days Since First Measurement
%Pred FEV1 correct
%Pred FEV1 incorrect
%Pred FVC correct
%Pred FVC incorrect

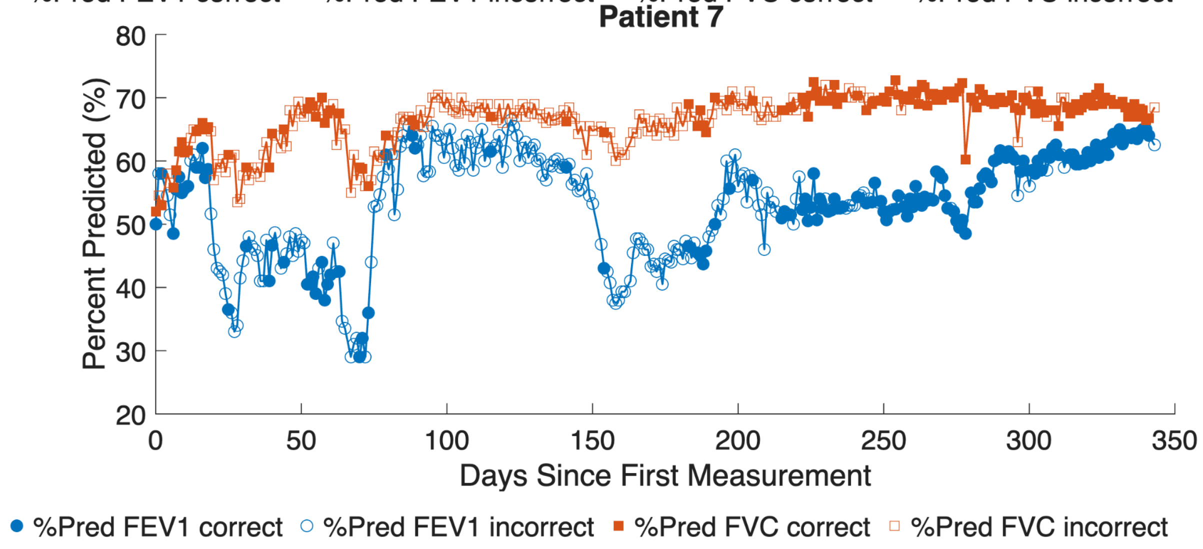
Patient 7
Percent Predicted (%)
80
70
60
50
40
30
20
0
50
100
150
200
250
300
350
Days Since First Measurement
%Pred FEV1 correct
%Pred FEV1 incorrect
%Pred FVC correct
%Pred FVC incorrect

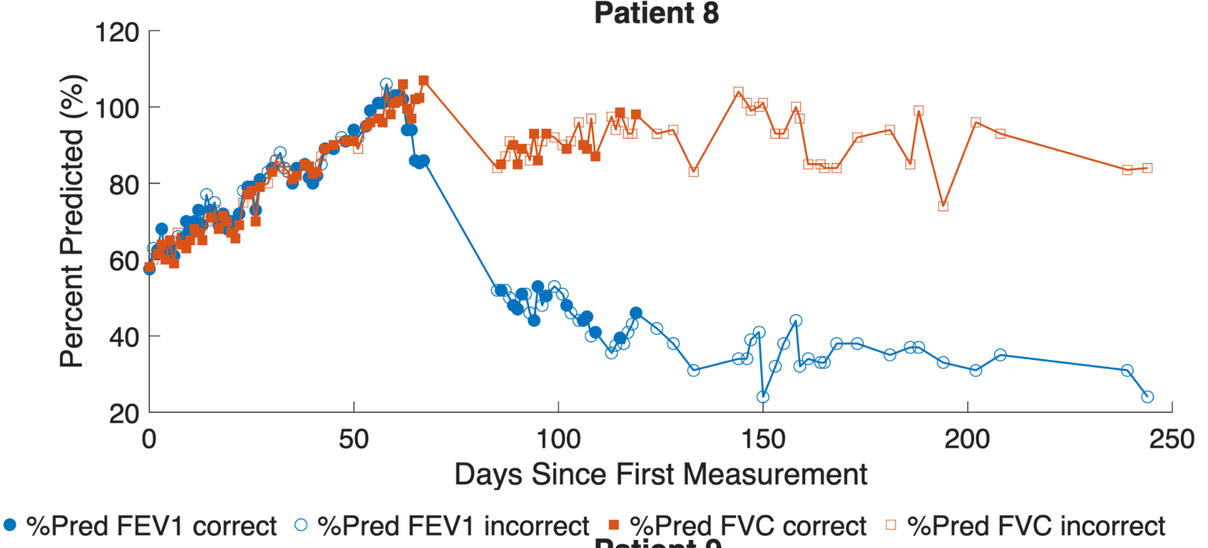
Patient 8
Percent Predicted (%)
Days Since First Measurement
%Pred FEV1 correct
%Pred FEV1 incorrect
%Pred FVC correct
%Pred FVC incorrect

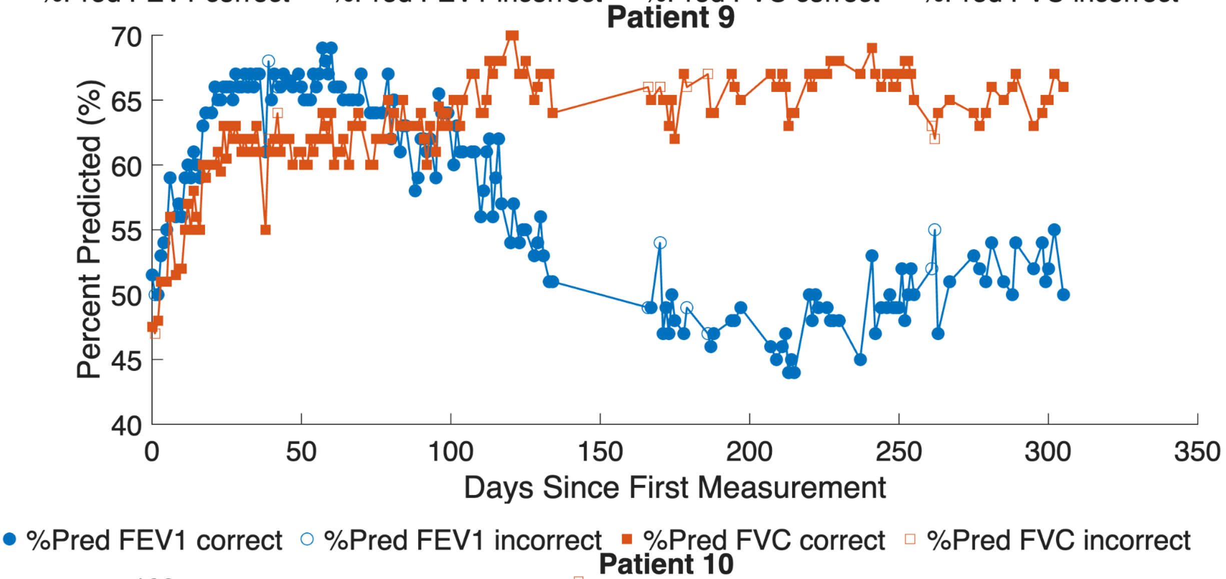
Patient 9
Percent Predicted (%)
Days Since First Measurement
%Pred FEV1 correct
%Pred FEV1 incorrect
%Pred FVC correct
%Pred FVC incorrect

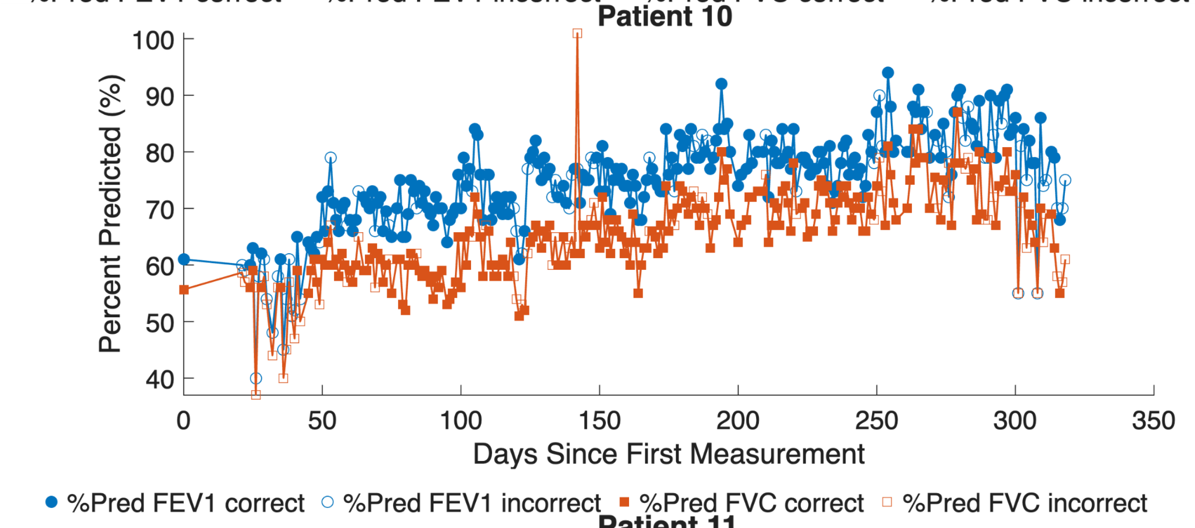
Patient 10
Percent Predicted (%)
Days Since First Measurement
%Pred FEV1 correct
%Pred FEV1 incorrect
%Pred FVC correct
%Pred FVC incorrect

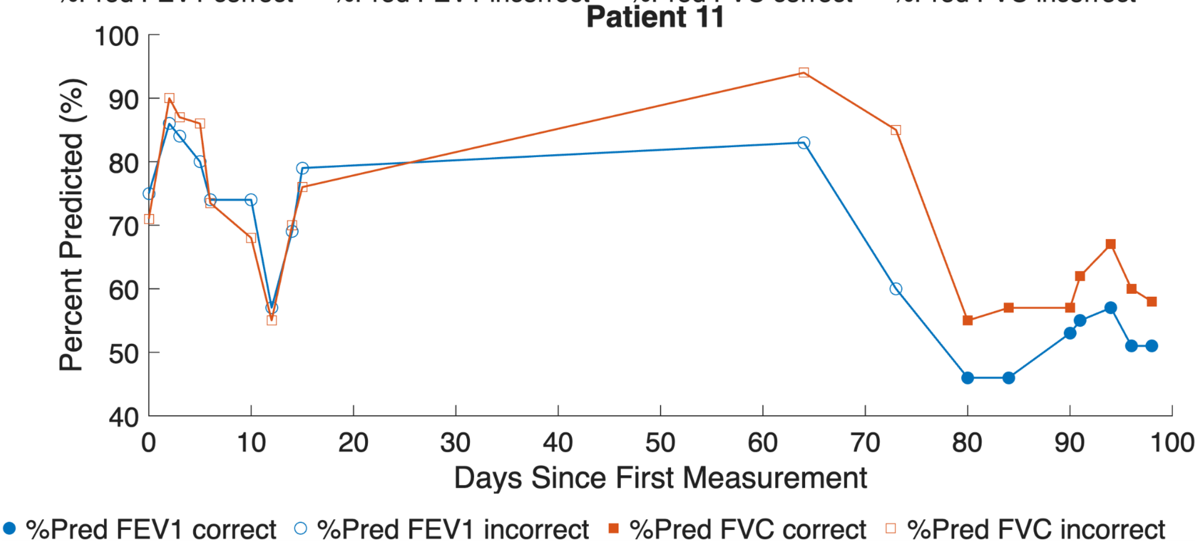
Patient 11
Percent Predicted (%)
Days Since First Measurement
%Pred FEV1 correct
%Pred FEV1 incorrect
%Pred FVC correct
%Pred FVC incorrect

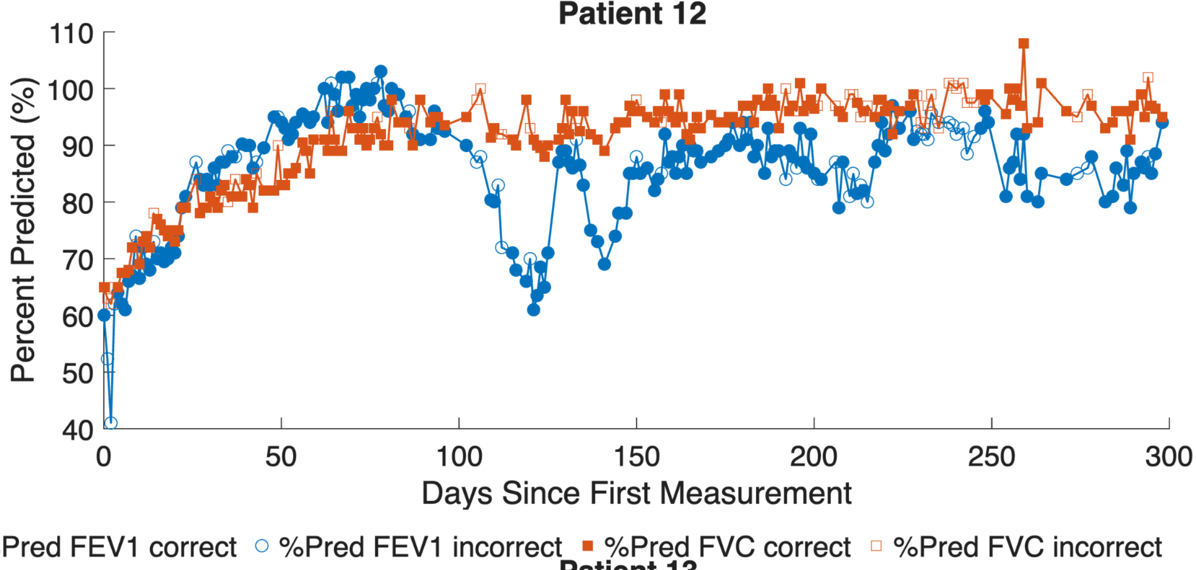
Patient 12
Percent Predicted (%)
Days Since First Measurement
%Pred FEV1 correct
%Pred FEV1 incorrect
%Pred FVC correct
%Pred FVC incorrect

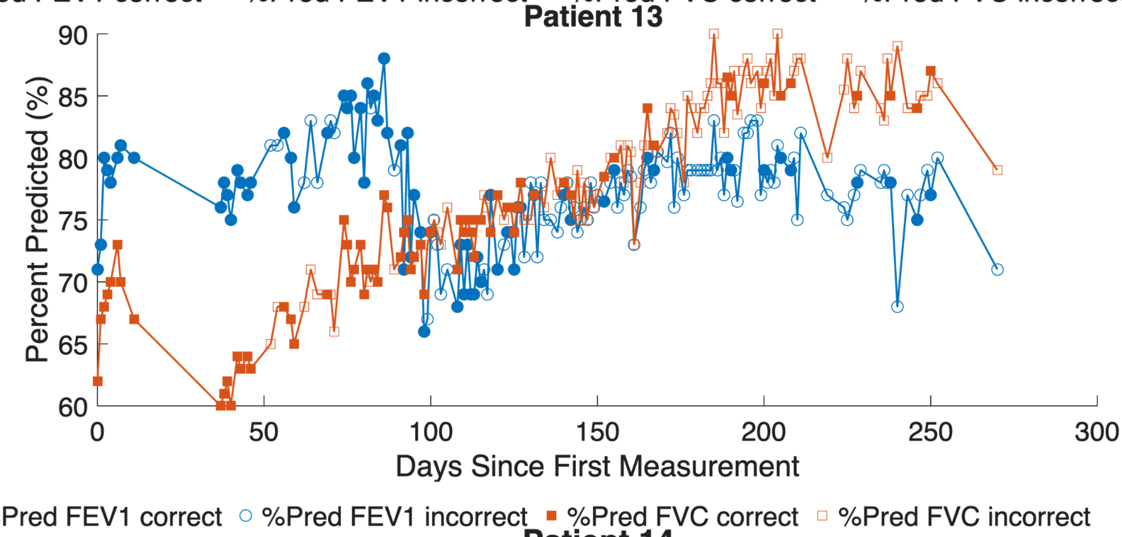
Patient 13
Percent Predicted (%)
Days Since First Measurement
%Pred FEV1 correct
%Pred FEV1 incorrect
%Pred FVC correct
%Pred FVC incorrect

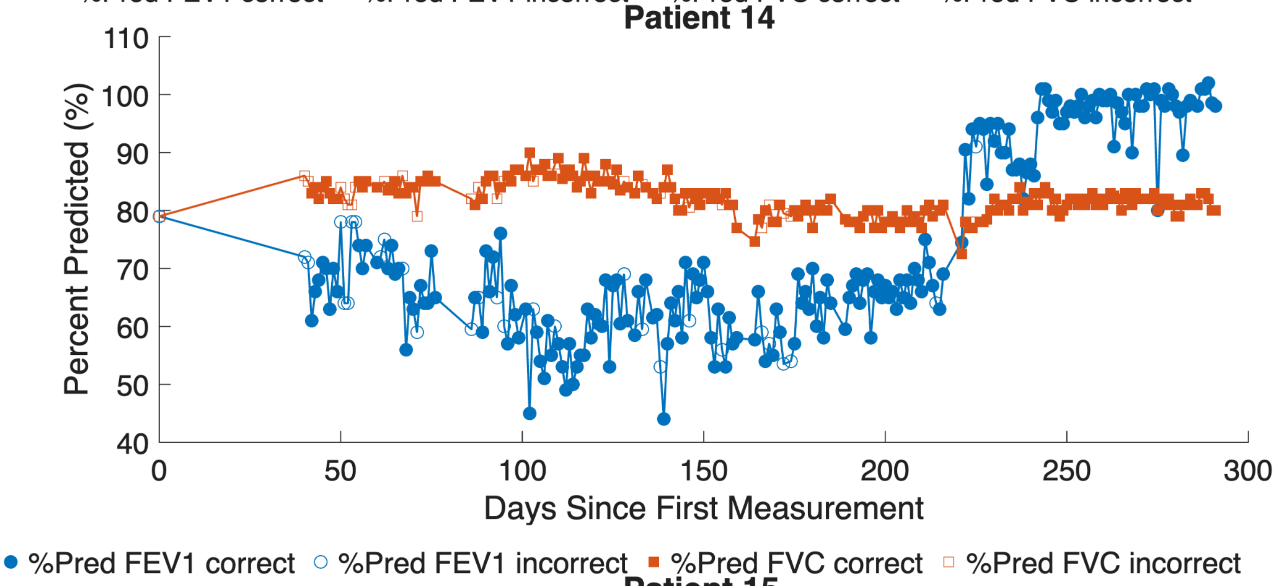
Patient 14
Percent Predicted (%)
Days Since First Measurement
%Pred FEV1 correct
%Pred FEV1 incorrect
%Pred FVC correct
%Pred FVC incorrect

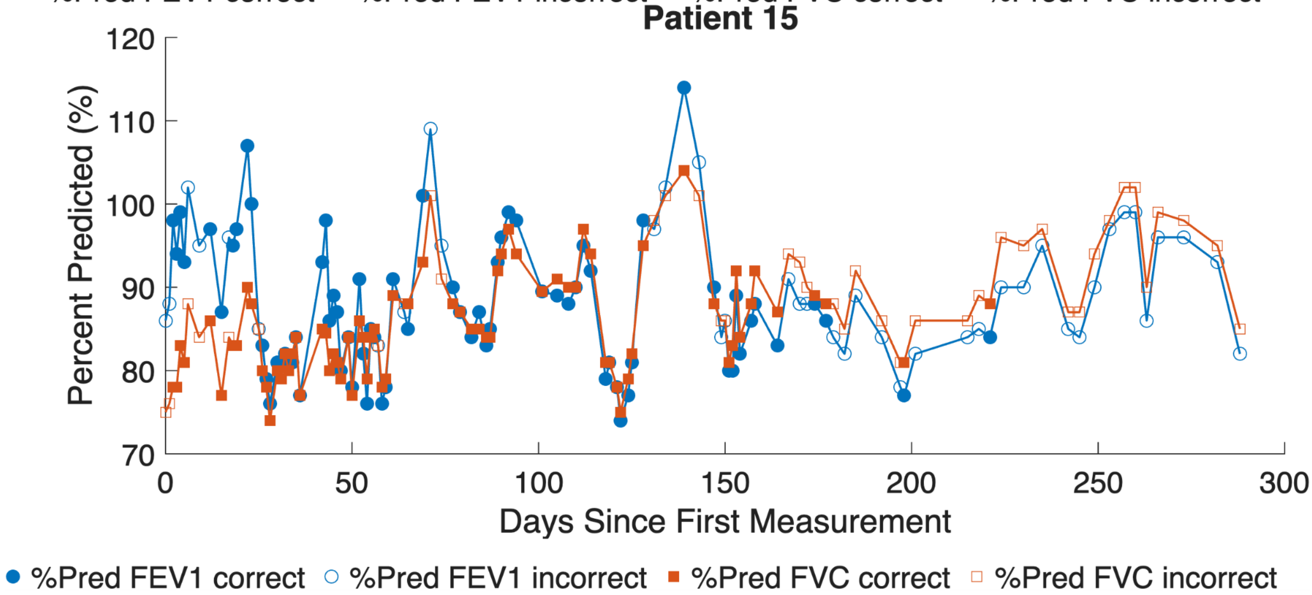
Patient 15
Percent Predicted (%)
Days Since First Measurement
%Pred FEV1 correct
%Pred FEV1 incorrect
%Pred FVC correct
%Pred FVC incorrect

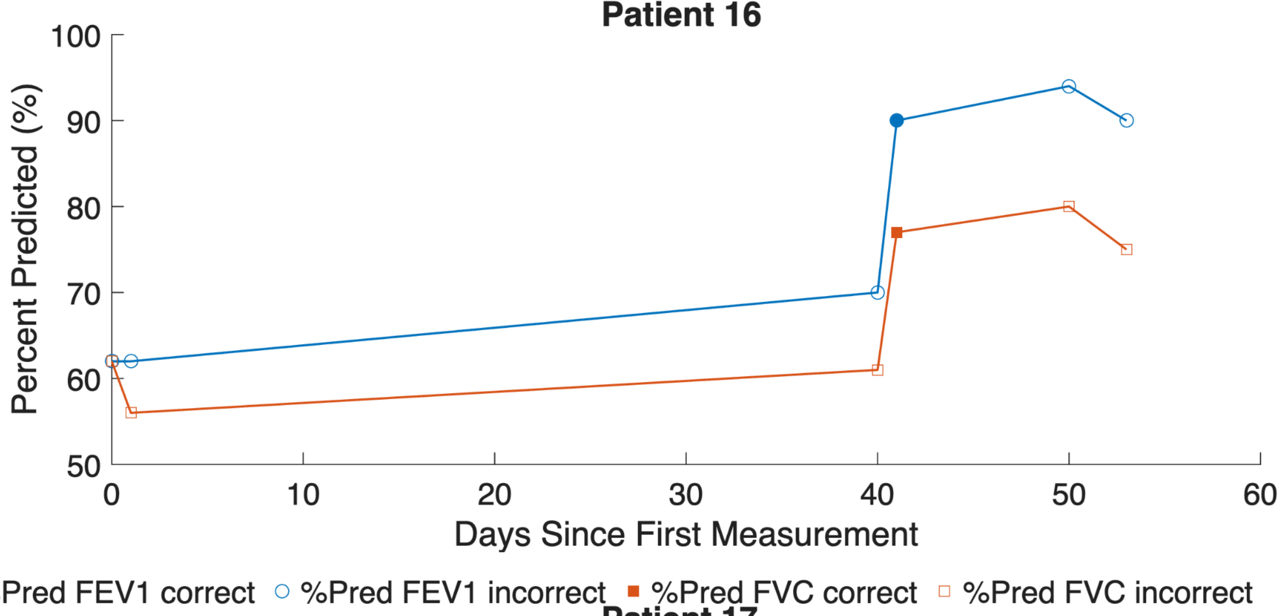
Patient 16
Percent Predicted (%)
100
90
80
70
60
50
0
10
20
30
40
50
60
Days Since First Measurement
%Pred FEV1 correct
%Pred FEV1 incorrect
%Pred FVC correct
%Pred FVC incorrect

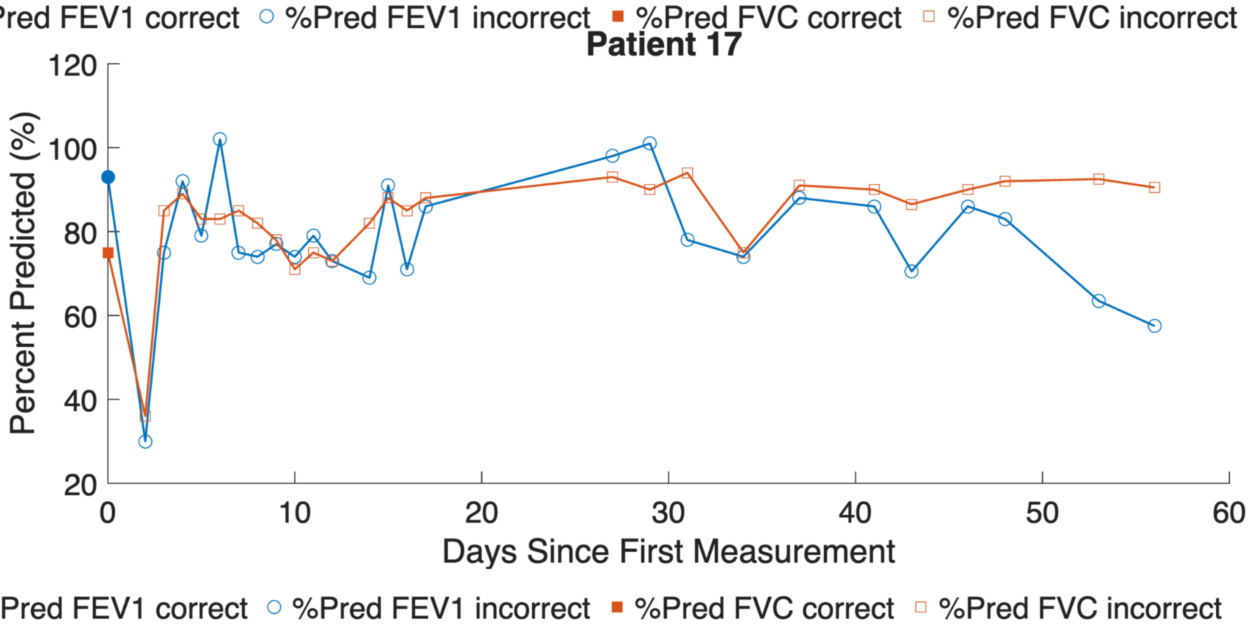
Patient 17
Percent Predicted (%)
120
100
80
60
40
20
0
10
20
30
40
50
60
Days Since First Measurement
%Pred FEV1 correct
%Pred FEV1 incorrect
%Pred FVC correct
%Pred FVC incorrect

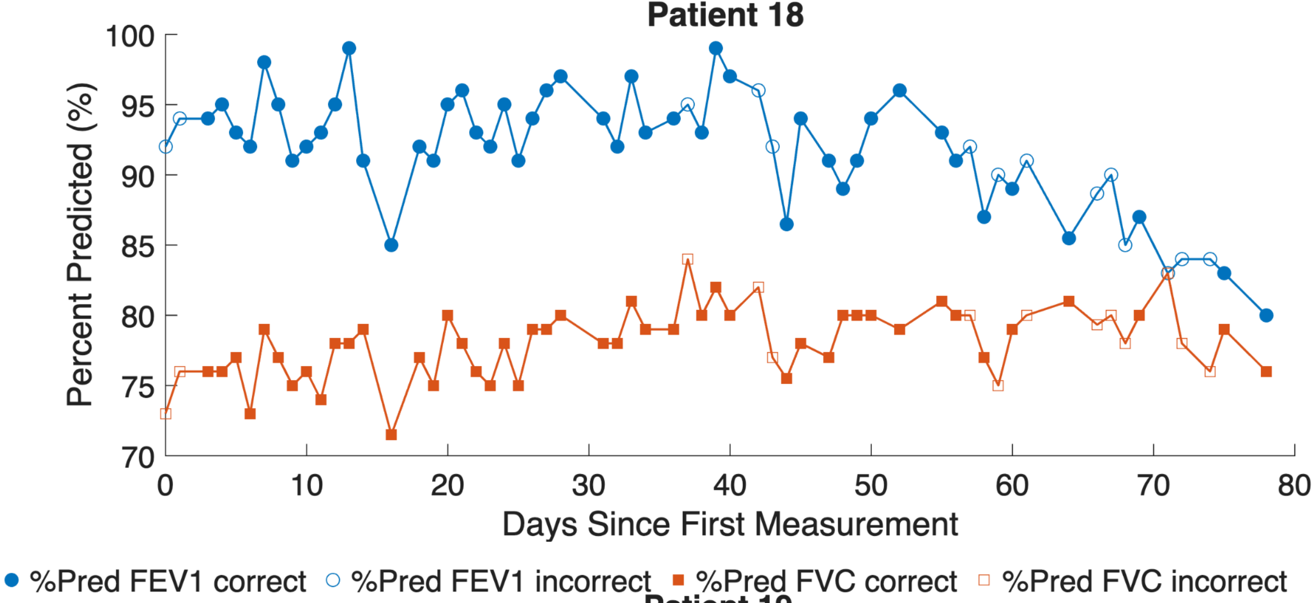
Patient 18
Percent Predicted (%)
100
95
90
85
80
75
70
0
10
20
30
40
50
60
70
80
Days Since First Measurement
%Pred FEV1 correct
%Pred FEV1 incorrect
%Pred FVC correct
%Pred FVC incorrect

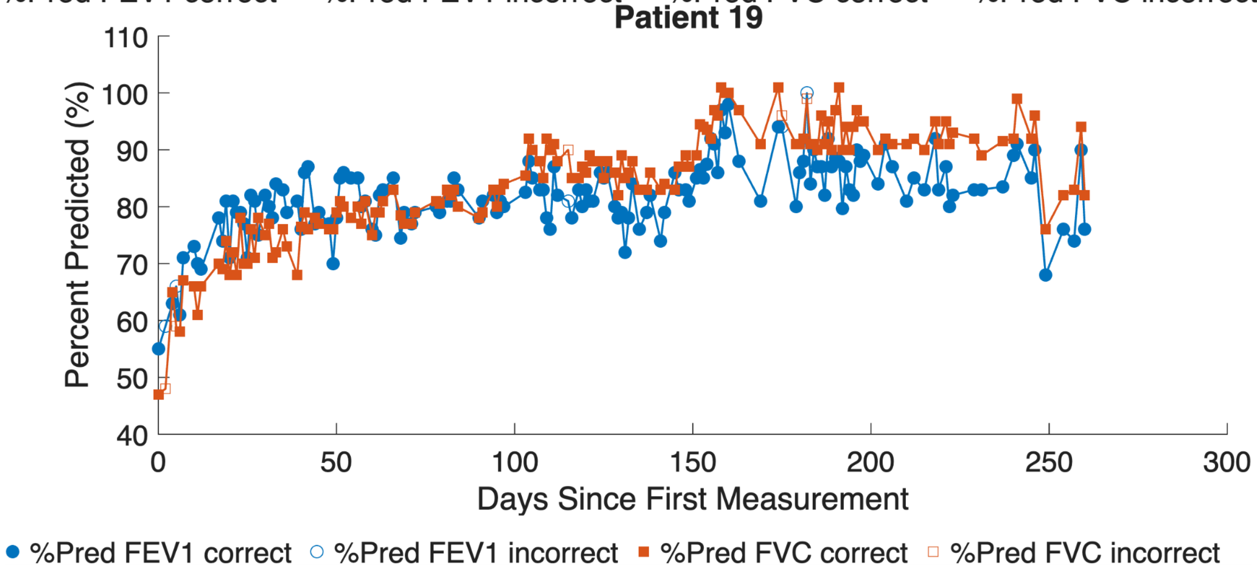
Patient 19
Percent Predicted (%)
110
100
90
80
70
60
50
40
0
50
100
150
200
250
300
Days Since First Measurement
%Pred FEV1 correct
%Pred FEV1 incorrect
%Pred FVC correct
%Pred FVC incorrect

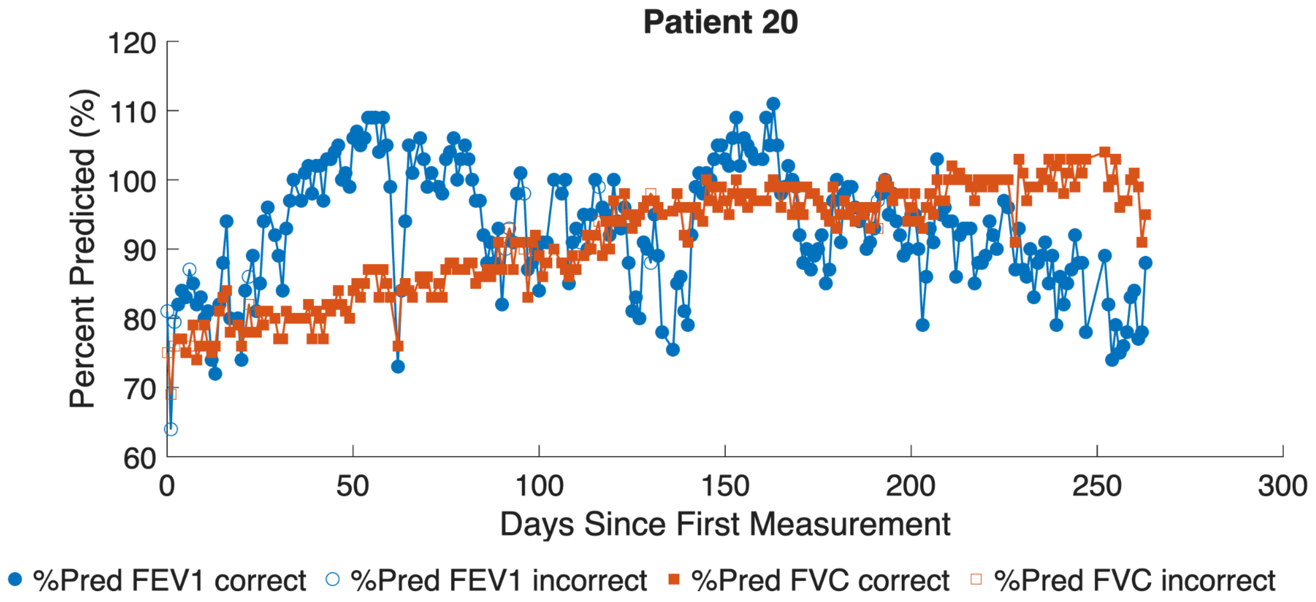
Patient 20
Percent Predicted (%)
120
110
100
90
80
70
60
0
50
100
150
200
250
300
Days Since First Measurement
%Pred FEV1 correct
%Pred FEV1 incorrect
%Pred FVC correct
%Pred FVC incorrect

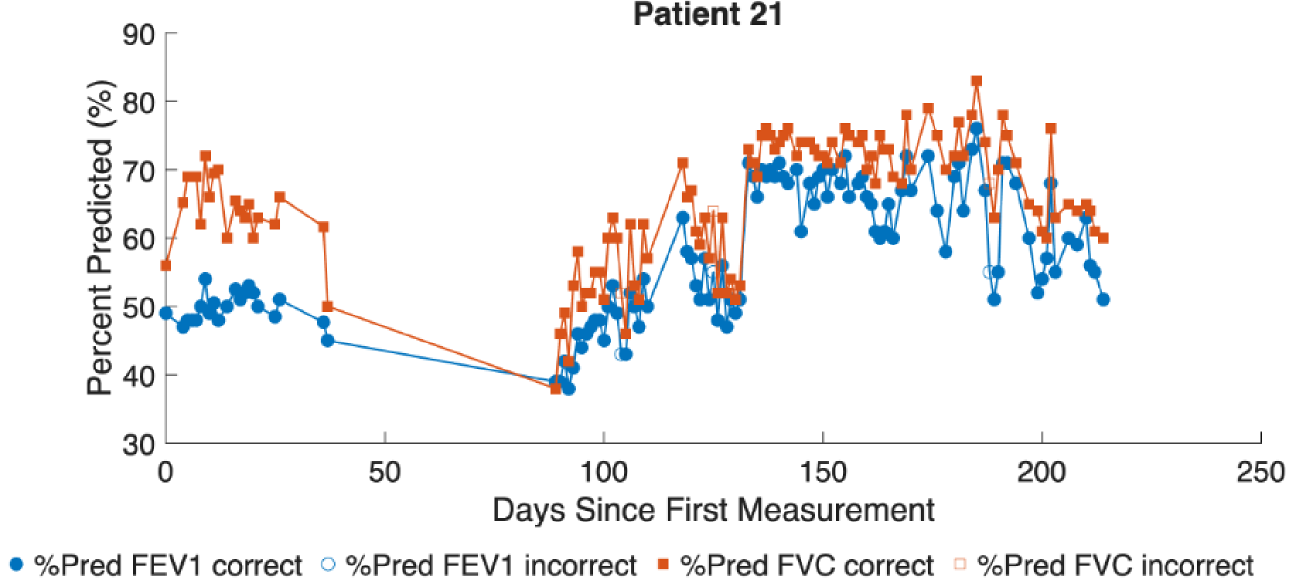

Mixed-models effect sizes

Table S2: Number of visits - All visits

Name	β	SE	tStat	DF	p-value	β Lower	β Upper
(Intercept)	0.17	2.59	0.07	70	0.946	-5.00	5.35
Age	0.00	0.03	-0.01	70	0.990	-0.06	0.06
Gender	-1.31	0.69	-1.88	70	0.064	-2.69	0.08
Observation duration	5.75	0.73	7.91	70	<0.001	4.30	7.20
TM group (rel. to RC)	1.81	2.73	0.66	70	0.509	-3.63	7.24
HM group (rel. to RC)	1.76	2.6	0.67	70	0.506	-3.493	7.014

Table S3: Number of visits - Planned visit

Name	β	SE	tStat	DF	p-value	β Lower	β Upper
(Intercept)	3.62	0.94	3.87	70	0.000	1.75	5.49
Age	-0.0014	0.0047	-0.29	70	0.770	-0.01	0.01
Gender	-0.57	0.49	-1.16	70	0.251	-1.54	0.41
Observation duration	3.62	0.38	9.44	70	<0.001	2.85	4.38
TM group (rel. to RC)	0.76	0.62	1.23	70	0.224	-0.48	1.99
HM group (rel. to RC)	0.19	0.6	0.32	70	0.750	-0.976	1.348

Table S4: Number of visits - Urgent visit

Name	β	SE	tStat	DF	p-value	Lower	Upper
(Intercept)	3.17	1.33	2.38	70	0.020	0.52	5.81
Age	-0.03	0.02	-1.21	70	0.229	-0.07	0.02
Gender	-0.48	0.49	-0.98	70	0.332	-1.45	0.50
Observation duration	0.54	0.36	1.51	70	0.136	-0.18	1.26
TM group (rel. to RC)	-0.88	0.58	-1.53	70	0.130	-2.03	0.27
HM group (rel. to RC)	-0.71	0.5	-1.29	70	0.201	-1.81	0.39

Table S5: Mean duration of visits - All visits

Name	β	SE	tStat	DF	p-value	β Lower	β Upper

(Intercept)	24.37	4.28	5.69	70	0.000	15.83	32.91
Age	-0.12	0.07	-1.86	70	0.067	-0.26	0.01
Gender	3.04	1.58	1.93	70	0.058	-0.10	6.19
Observation duration	-6.45	1.16	-5.54	70	<0.001	-8.77	-4.12
TM group (rel. to RC)	-7.14	1.86	-3.84	70	<0.001	-10.84	-3.43
HM group (rel. to RC)	-3.35	1.77	-1.89	70	0.063	-6.88	0.19

Table S6: Mean duration of visits - Planned visit

Name	β	SE	tStat	DF	p-value	β Lower	β Upper
(Intercept)	10.49	5.34	1.97	70	0.053	-0.16	21.13
Age	-0.04	0.08	-0.43	70	0.667	-0.20	0.13
Gender	3.94	1.97	2.00	70	0.049	0.02	7.86
Observation duration	-4.13	1.45	-2.85	70	0.006	-7.02	-1.24
TM group (rel. to RC)	-3.35	2.32	-1.45	70	0.152	-7.97	1.27
HM group (rel. to RC)	2.30	2.2	1.04	70	0.301	-2.10	6.71

Table S7: Mean duration of visits - Urgent visit

Name	β	SE	tStat	DF	p-value	β Lower	β Upper
(Intercept)	34.45	9.06	3.80	70	0.000	16.38	52.52
Age	0.05	0.14	0.36	70	0.717	-0.23	0.33
Gender	1.93	3.34	0.58	70	0.565	-4.73	8.58
Observation duration	-8.29	2.46	-3.37	70	0.001	-13.20	-3.38
TM group (rel. to RC)	-15.10	3.93	-3.84	70	<0.001	-22.94	-7.26
HM group (rel. to RC)	-14.17	3.7	-3.78	70	<0.001	-21.65	-6.70

Table S8: Max duration of visits - All visits

Name	β	SE	tStat	DF	p-value	β Lower	β Upper
(Intercept)	68.00	15.46	4.40	70	0.000	37.16	98.83
Age	-0.37	0.23	-1.59	70	0.117	-0.84	0.10
Gender	8.76	5.56	1.58	70	0.120	-2.33	19.85
Observation duration	-8.70	4.44	-1.96	70	0.054	-17.57	0.16
TM group (rel. to RC)	-14.61	8.82	-1.66	70	0.102	-32.21	2.98
HM group (rel. to RC)	-11.24	7.96	-1.41	70	0.163	-27.12	4.64

Table S9: Mean duration of visits - Planned visit

Name	β	SE	tStat	DF	p-value	β Lower	β Upper
(Intercept)	23.85	11.05	2.16	70	0.034	1.81	45.89
Age	-0.10	0.17	-0.60	70	0.550	-0.44	0.24
Gender	7.86	4.03	1.95	70	0.055	-0.18	15.89
Observation duration	-5.68	3.11	-1.83	70	0.072	-11.87	0.52
TM group (rel. to RC)	-3.65	5.66	-0.64	70	0.521	-14.94	7.64
HM group (rel. to RC)	4.51	5.16	0.87	70	0.386	-5.79	14.81

Table S10: Mean duration of visits - Urgent visit

Name	β	SE	tStat	DF	p-value	β Lower	β Upper

(Intercept)	52.45	15.60	3.36	70	0.001	21.33	83.56
Age	-0.27	0.22	-1.20	70	0.233	-0.72	0.18
Gender	1.69	5.28	0.32	70	0.751	-8.85	12.22
Observation duration	-5.66	4.65	-1.22	70	0.227	-14.93	3.61
TM group (rel. to RC)	-13.59	11.14	-1.22	70	0.227	-35.80	8.62
HM group (rel. to RC)	-14.50	10.2	-1.42	70	0.159	-34.82	5.82
